# Bioactive Glycosylated Flavonoids Exhibiting LXR Agonist Activity from a Lauraceae Colombian Species

**DOI:** 10.3390/plants14213240

**Published:** 2025-10-22

**Authors:** Juanita Pulido-Teuta, Fabian López-Vallejo, Adrián G. Sandoval-Hernández, Carlos-Eduardo Narváez-Cuenca, Mónica Avila-Murillo

**Affiliations:** 1 QuiProNaB, Departamento de Química, Facultad de Ciencias, Universidad Nacional de Colombia, Sede Bogotá, Carrera 30 No 45-03, Bogotá 111321, Colombia; jpulitot@unal.edu.co (J.P.-T.); fhlopezv@unal.edu.co (F.L.-V.); 2 Grupo de Muerte Celular, Instituto de Genética, Universidad Nacional de Colombia, Sede Bogotá, Carrera 30 No 45-03, Bogotá 111321, Colombia; agsandovalh@unal.edu.co; 3 Food Chemistry Research Group, Departamento de Química, Facultad de Ciencias, Universidad Nacional de Colombia, Sede Bogotá, Carrera 30 No 45-03, Bogotá 111321, Colombia; cenarvaezc@unal.edu.co

**Keywords:** liver X receptors, inflammatory response, *Nectandra*, glycosylated flavonoids, molecular docking, agonistic activity

## Abstract

Lipid metabolism is a vital biological process essential for human health, encompassing key pathways necessary for the survival and homeostasis of all organisms. Liver X Receptors (LXRs) are extensively acknowledged as pivotal regulators of lipid homeostasis and inflammatory responses. Pharmacological activation of Liver X Receptor (LXR) has been shown to increase expression of ApoE and ABCA1 proteins, reducing neurodegeneration in murine models of Alzheimer’s disease. Because previous reports determined that *Nectandra reticulata* (Lauraceae) extract has agonistic LXRs activity, the objective of this study was to determine the metabolites present in this extract and to evaluate their in silico and in vitro agonistic activity. The chromatographic analysis revealed the presence of three glycosylated flavonols. The in silico study showed that isolated flavonoids generate a hydrogen bond with T302 and T316 (LXRα and LXRβ, respectively). The in vitro study showed that the flavonoids increased the expression of mRNA of both *APOE* and *ABCA1* target genes of LXRs, as observed by qRT-PCR. The bioactive flavonoids isolated in this study possess a documented antioxidant effect; when combined with their LXR agonist activity, they become promising bioactive candidates for use in nutraceutical formulations aimed at promoting brain health and anti-inflammatory effects.

## 1. Introduction

Liver X Receptors (LXRs) have gained increasing attention in Alzheimer’s disease (AD) research for their central role in regulating cholesterol homeostasis and inflammation—two key processes in AD pathogenesis [1,2]. Activation of LXRs by agonists modulates Apolipoprotein E (APOE) and the ABCA1 transporter, promoting HDL formation, reducing amyloid-beta (Aβ) production, and enhancing its clearance [3,4,5,6,7]. Their anti-inflammatory effects further support microglial-mediated removal of neurotoxic Aβ aggregates, reinforcing their potential as therapeutic targets for AD [4]. With this in mind, metabolites with LXR agonist action may be candidates for more advanced studies if therapeutic options for AD are sought.

Previous studies carried out by our research group have shown LXR agonist activity of *Nectandra reticulata* leaf extract [8]. We already reported the presence of quercitrin in a *Nectandra reticulata* leaf extract, a compound that was identified by means of the comparison of retention time and UV-vis maxima of a chromatographic signal against those of an authentic standard [9]. In the mentioned work, neither further identification strategies were attempted for quercitrin nor for other metabolites. Aside from that report, no additional information on the metabolites of this species is currently available; however, studies on metabolites have been conducted for other species within the genus. Some of the metabolites described for the *Nectandra* genus are flavonoids, lignoids, phenylpropanoids, steroids, terpenoids, and alkaloids [10,11]. Many of them exhibit anti-inflammatory, anti-fatty liver disease, and antioxidant activities.

Quercitrin positively influences key biological pathways involved in the progression of AD, with well-documented antioxidant and free radical scavenger activity [12], as well as the ability to inhibit acetylcholinesterase. Besides that, quercitrin reduces the accumulation of amyloid-β plaques in AD model mice [13]. Considering that the leaf extract of *N. reticulata* has LXR agonist activity and that its metabolic profile has not fully been characterized, this study aimed to characterize the flavonoid profile of a *N. reticulata* ethanolic extract to confirm the presence of quercitrin, to identify additional metabolites, and to assess their potential as Liver X Receptor agonists through in silico molecular docking and in vitro gene expression assays using real-time PCR. The results of this integrative study offer a more robust structural basis for the observed in vitro upregulation of APOE and ABCA1, thereby supporting the therapeutic potential of glycosylated flavonoids from *Nectandra reticulata* in modulating lipid metabolism and promoting neuroprotection.

## 2. Results and Discussion

In this research, an ethanolic extract from *Nectandra reticulata* was analyzed to identify its most abundant compounds and to test them for in silico LXR agonist activity, as well as their in vitro agonist activity of both *APOE* and *ABCA1* target genes of LXRs. Three compounds were found as the main representatives: quercitrin (quercetin-3-*O*-rhamnoside), afzelin (kaempferol-3-*O*-rhamnoside), and either kaempferol-3-*O*-(6″-*p*-coumaroylglucoside) or kaempferol-7-*O*-(6″-*p*-coumaroylglucoside). Despite high-resolution mass spectrometry data being crucial to annotate such compounds, acidic hydrolysis was useful to rule out if, e.g., kaempferol or luteolin were part of a compound, since both aglycones have the same molecular weight. When performing such acid hydrolysis, a special emphasis was placed to find the conditions in which the compound was successfully hydrolyzed while ensuring the released constituents remained stable and soluble [14]. Furthermore, the identified compounds were found to have LXR agonistic activity as shown not only by the degree of the interaction between them and the amino acids threonine 302 and threonine 316 (LXRα and LXRβ, respectively) but also because of the increased expression of mRNA of both *APOE* and *ABCA1* target genes of LXRs as induced by the presence of such isolated compounds.

### 2.1. Identification of the Metabolites

**Figure 1:**
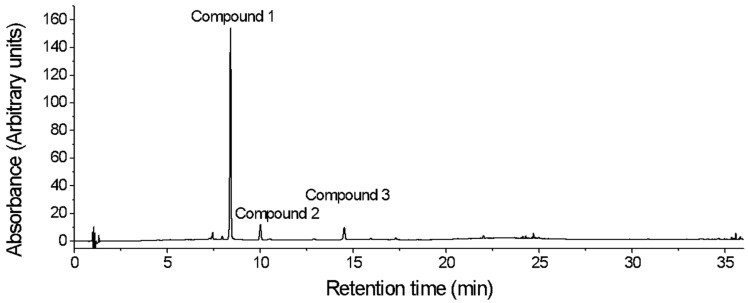
Chromatographic profile of the ethanolic extract of *Nectandra reticulata* as recorded at 355 nm.

The chromatographic profile of *Nectandrareticulata* crude extract showed three compounds with characteristic UV-vis spectra of glycosylated flavonoids (Table 1) [15]. Those three compounds accounted for 99.35% (Figure 1) of the chromatographic area recorded at 355 nm. UV-vis spectra of flavonoids are typically characterized by two absorption maxima (that is, Band II and Band I) [16]. Depending on the wavelength of the maxima, different types of flavonoids can be differentiated. The maximum UV observed for compounds **1**–**3** at 260–268 nm and at 314–349 nm (Table 1) is characteristic of either flavones or flavonols [17]. From the literature, it is known that these types of flavonoids show Band I between 300 and 380 nm and Band II between 240 and 280 nm, with hypsochromic shifts if they are either glycosylated or methylated [16]. Additionally, the molecular ions of the compounds **1**–**3** ([M+H]^+^ or [M−H]^−^) together with the daughter ions can be found in Table 1 as well. Given that the molecular masses of flavones and flavonols typically range between 220 and 301 a.m.u. [18], the higher molecular masses detected for compounds **1**–**3** indicate that these molecules likely correspond to flavones or flavonols carrying glycosidic moieties or bearing additional structural substitutions.

Compound 1, with a retention time of 7.80 min, was the most abundant with a relative area of 84.46% (Figure 1). This compound had two absorbance maxima at 260 and 349 nm and showed a molecular ion in negative ion mode at *m*/*z* 447.0936 ([M−H]^−^) (Table 1). The fragmentation of the molecular ion peak showed the detachment of a rhamnosyl fragment, thus yielding an aglycone at *m*/*z* 300.0273, which is likely to be a product of an homolytic fragmentation of the parent ion, in agreement with previous reports on the fragmentation of quercitrin isolated from the pericarp of *Juglans nigra* [19].

**Figure 2:**
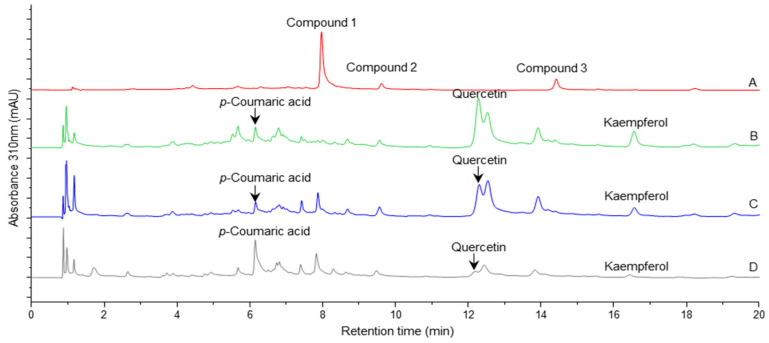
Chromatographic profile of the different hydrolysates as recorded at 310 nm between 0 and 20 min. (A) Non-hydrolyzed extract. (B) Hydrolysis at 80% (*v*/*v*) methanol. (C) Hydrolysis at 75% (*v*/*v*) methanol. (D) Hydrolysis at 50% (*v*/*v*) methanol. Following hydrolysis, multiple chromatographic peaks were detected. Among these, *p*-coumaric acid, quercetin, and kaempferol were identified by comparison with authentic standards, whereas the other peaks generated during hydrolysis remain uncharacterized.

**Figure 3:**
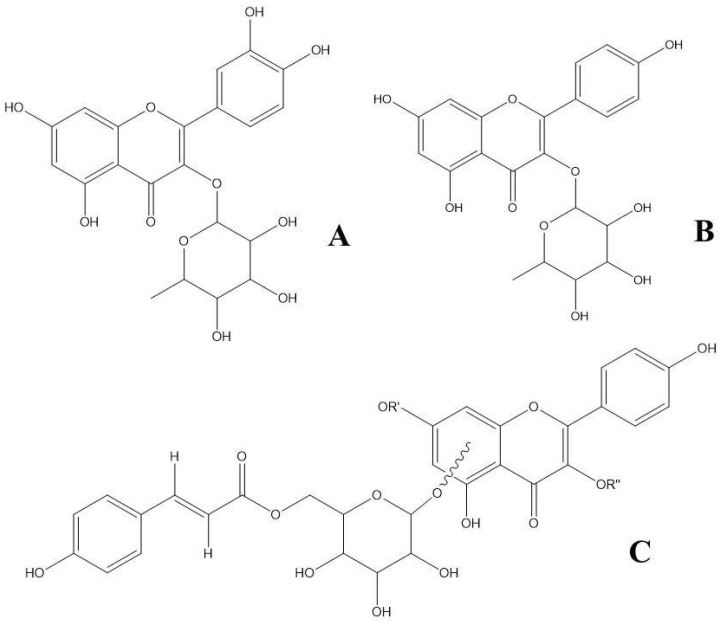
Chemical structure of the marker compounds (**A**) quercitrin [quercetin-3-rhamnoside], (**B**) afzelin [(kaempferol-3-*O*-rhamnoside], (**C**) kaempferol-3-*O*-(6″-*p*-coumaroylglucoside) or kaempferol-7-*O*-(6″-*p*-coumaroylglucoside).

In positive ion mode, no signal corresponding to the molecular ion was observed; instead, a signal corresponding to the aglycone was present at *m*/*z* 303.0509 [M-rhamnose+H]^+^. These signals suggest that compound **1** has quercetin as a flavonol in its structure. When the *N. reticulata* crude extract was hydrolyzed, the aglycone quercetin appeared as the most abundant reaction product (Rt at 12.29 min, UVmax at 257 and 368 nm; chromatographic and spectroscopic data similar to those of authentic quercetin) (Figure 2 and Table 2). It was concluded, therefore, that compound **1** corresponded to quercitrin (quercetin-3-*O*-rhamnoside) (Figure 3A).

Compound **2** in the crude extract of *Nectandra reticulata* was found at a retention time of 9.44 min, with absorbance maxima at 260 and 341 nm and a relative area of 8.00% (Figure 1). It showed a molecular ion peak at *m*/*z* 431.0980 in negative ion mode. Fragmentation of that parent ion yielded an ion at *m*/*z* 284.0323, as a result of a loss of rhamnose ([M-rhamnose-2H]^−●^) [20]. In contrast, in positive ion mode, the molecular ion peak was not found; only the loss of a sugar moiety to yield the aglycone at *m*/*z* 287.0561 [M-rhamnose+H]^+^ was observed. The ion at *m*/*z* 284.0323 in negative ion mode, together with the ion in the positive mode at *m*/*z* 287.0561 and the UV maxima, are indicative of the presence of either kaempferol or luteolin in the structure of compound **2**. When hydrolyzing the crude extract, the aglycone kaempferol was found (retention time and UV maxima of the released aglycone equal to those of authentic kaempferol). Therefore, it was concluded that compound **2** corresponded to afzelin (kaempferol-3-*O*-rhamnoside). The fragmentation pattern of afzelin found here, including its homolytic cleavage, is consistent with previous reports of this compound when extracted from stems of *Ephedra gerardiana* [21]. Both compounds **1** and **2** have been previously reported in other species of the *Nectandra* genus, such as *Nectandra glabrescens*, *Nectandra grandiflora*, and *Nectandra amazonum* [20,22,23,24]. Furthermore, compound **1** was previously reported by us in an extract of *N. reticulata* [9].

Compound **3** had a retention time of 14.26 min, UV-VIS maxima at 268 and 314 nm, and a relative area of 6.89% (Figure 1). This compound, in positive ion mode, exhibited a signal of the molecular ion peak and a loss of a coumaroyl glucoside at *m*/*z* 595.1464 [M+H]^+^ and *m*/*z* 287.0559 [M-coumaroyl glucoside+H]^+^, respectively. In negative ion mode, compound **3** revealed the ions at *m*/*z* 593.1296 [M−H]^−^ and at *m*/*z* 285.0396 [M-coumaroyl glucoside-H]^−^. Additionally, in positive ion mode, it yielded a signal at *m*/*z* 309.0981 [M-kaempferol+H]^+^ that is common in metabolites that have a coumaroyl glucoside decoration in their structure [25]. Daughter ions, in both negative and positive ion modes, showed that there is a flavonoid with a mass of 286 a.m.u. in the structure. As mentioned above, the only possible aglycone (product of the hydrolysis reaction) is kaempferol. Furthermore, the presence of *p*-coumaric acid was verified in the hydrolyzed extract by comparison with the authentic standard. With these analysis conditions, however, it was not possible to state if the *p*-coumaroyl glucoside substitution is in the 3- or 7- position of the flavonoid scaffold. Studies of the genus *Nectandra* have shown the presence of some kampferol derivatives, glycosilated in position 3- such as kaempferol-3-*O*-*α*-rhamnopyranoside and kaempferol-3-*O*-*α*-(3,4-di-E-*p*-coumaroyl)-rhamnopyranoside [26]. Some studies of the Lauraceae family indicate that most of the elucidated compounds have the substitution in position 3- of the flavonoid nucleus; in fact, the flavonoids quercetin and kaempferol are the most common flavonoids [10,27]. If the biosynthesis of glycosylated flavonoids in plants is considered, it is known that the most common substitution is also at carbon 3 of flavonoid [28,29]. It is also known that there are enzymes, such as UDP-flavonoid-3-*O*-glucosyltransferase, that exhibit strict regio-selectivity for position 3 [30]. This information together allows us to conclude that compound **3** is more likely to be kaempferol-3-*O*-(6″-*p*-coumaroylglucoside) rather than kaempferol-7-*O*-(6″-*p*-coumaroylglucoside). Fragmentation patterns in both negative and positive ion modes are consistent with previous reports on the fragmentation pattern of kaempferol-3-*O*-(6″-*p*-coumarylglucoside) from extracts of *Fagonia arabica* L. [31].

Quercitrin, found in the extract of *Nectandra reticulata*, has a wide spectrum of pharmacological activities including antioxidative stress, anti-inflammation, anti-microorganisms, immunomodulation, analgesia, wound healing, and vasodilation. In addition, several studies have tentatively suggested that quercitrin may contribute to the management of various health conditions, such as osteoporosis, osteoarthritis, gastrointestinal disorders, and neurodegenerative diseases [32]. Afzelin and kaempferol-3-*O*-(6″-*p*-coumaroylglucoside), despite being present in relatively lower abundance than quercitrin in the extract of *Nectandra reticulata*, are also considered compounds of significant interest. On the one hand, afzelin has emerged as a compound with considerable therapeutic interest due to its anti-inflammatory, antioxidative, anti-cancer, and neuroprotective effects [33]. On the other hand, kaempferol-3-*O*-(6″-*p*-coumaroylglucoside) has been reported to exhibit anti-diabetic [34], anti-cancer, and antibiotic effects [35].

### 2.2. Evaluation of Hydrolysis Conditions

Identification presented in the previous section required the confirmation of the aglycones by hydrolysis. Acidic hydrolysis of the crude extract allowed us not only to identify the aglycones of the glycosylated flavonoids present in the mixture but also revealed an important effect of the composition of the hydrolysis solvent mixture. To confirm the presence of quercetin, *p*-coumaric acid, and to rule out the presence of either luteolin or kaempferol, authentic standards were analyzed under the same chromatographic conditions that the crude extract and the hydrolysates were studied.

When hydrolysis was performed with either 50 or 75% (*v*/*v*) methanol, a complete hydrolysis of compound **1** was not observed (Figure 2). Instead, when performed at 80% (*v*/*v*) methanol, compound **1** was fully hydrolyzed. Furthermore, while compound **2** was not fully hydrolyzed at either of the tested methanol concentrations, compound **3** was hydrolyzed the most at 50% (*v*/*v*) methanol. As a result of the hydrolysis process, the area of the released quercetin and kaempferol increased when using 80% (*v*/*v*) methanol. In contrast, a greater chromatographic area was found for *p*-coumaric acid when hydrolysis was carried out with 50% (*v*/*v*) methanol. Under the tested conditions, luteolin was not detected. We hypothesize that the variation in the chromatographic area of quercetin, kaempferol, and *p*-coumaric acid with different methanol concentration reflects differences in the extent of the hydrolysis, the stability of the released aglycones, and the solubility of the released compounds under the hydrolysis conditions. Based on previous reports, the hydrolysis conditions employed in this study preserve both the stability of flavonols and the efficiency of the extraction process. Controlled temperature, and suitable solvents such as 50% aqueous methanol, prevents the formation of acid-induced degradation products of flavonols, including chalcones. Furthermore, the incorporation of small amounts of ascorbic acid provides antioxidant protection, thereby minimizing the degradation of aglycones generated during hydrolysis [36].

### 2.3. Molecular Docking into the Agonist Binding Sites of LXRα and LXRβ Receptors

Previous studies reported the agonist activity of different flavonoids against LXRs [37], including cyanidin [38], quercetin, and apigenin [15,30]. A previously detailed study indicated that the hydrogen bond with threonine 302 and threonine 316 (LXRα and LXRβ, respectively) would be involved in the agonist activity of the studied compounds. Because of that, re-docking was performed with the protein data bank (PDB) files for hLXRα-LBD (3IPQ) and hLXRβ-LBD (1PQ6) and the crystal compound GW3965 to ensure that the conditions of the grid box and docking parameters were able to reproduce the binding modes as reported in the literature [15].

**Figure 4:**
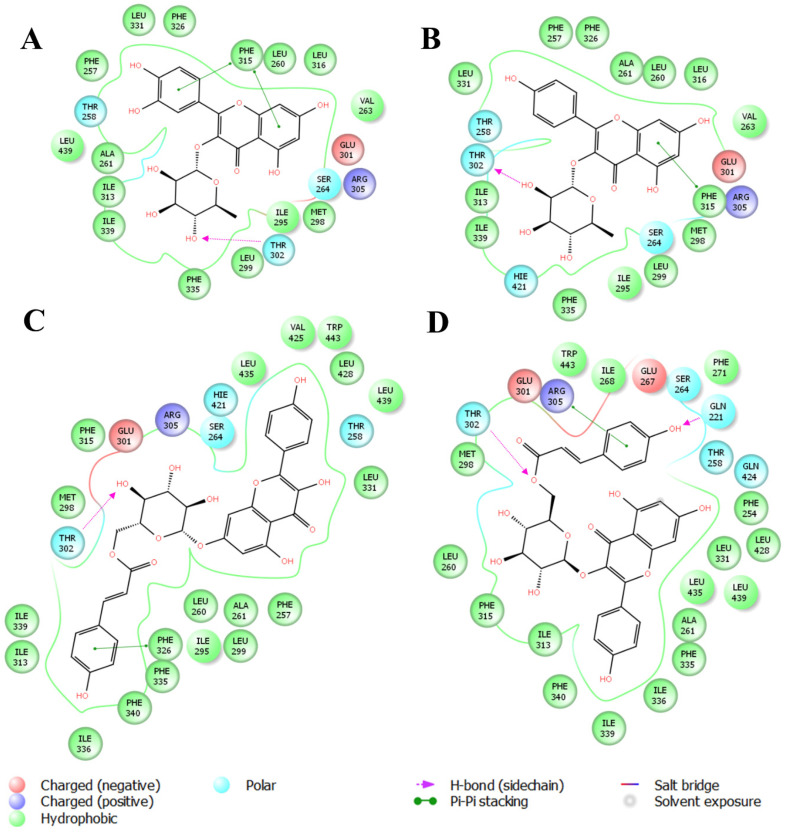
Binding modes of flavonoid compounds with LXRα (PDB: 3IPQ). (**A**) shows quercitrin, (**B**) afzelin, (**C**) kaempferol 7-(6″-*p*-coumaroylglucoside), and (**D**) kaempferol 3-(6″-*p*-coumaroylglucoside).

**Figure 5:**
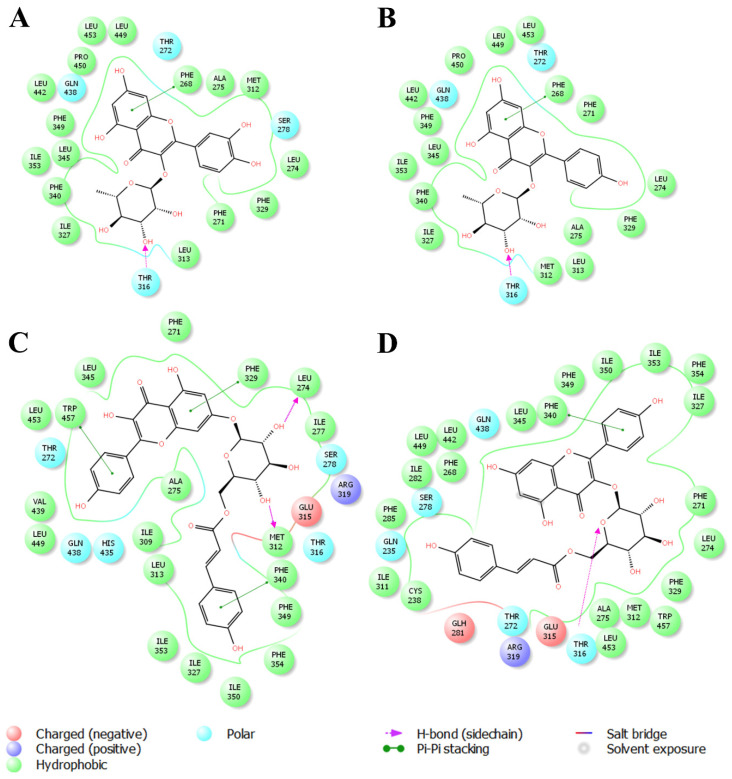
Binding modes of flavonoid compounds with LXRβ (PDB: 1PQ6). (**A**) shows quercitrin, (**B**) afzelin, (**C**) kaempferol 7-(6″-*p*-coumaroylglucoside), and (**D**) kaempferol 3-(6″-*p*-coumaroylglucoside).

Likewise, molecular docking of the natural flavonoids, quercetin and apigenin, was carried out. As expected from the work by Fouache et al. [15], an effective hydrogen bond for both LXRα and LXRβ was observed. After this, the binding modes of the compounds identified in the crude extract of *Nectandra reticulata* were predicted. The binding modes with the lowest energy are presented (Figure 4 and Figure 5). Because it was not possible to determine the substitution of the *p*-coumaroyl glucoside in compound **3**, both possibilities [kaempferol-3-O-(6″-*p*-coumaroylglucoside) and kaempferol-7-O-(6″-*p*-coumaroylglucoside)] were tested.

In addition to the canonical hydrogen bond with Thr302 (LXRα) and Thr316 (LXRβ), which has been consistently linked to agonist activity [15,39], our docking results revealed additional stabilizing interactions. For LXRα, quercitrin (compound **1**) and afzelin (compound **2**) also showed π–π stacking with Phe315, whereas kaempferol 7-O-(6″-*p*-coumaroylglucoside) (compound **3**) established π–π contacts with Phe326 and Arg305, as well as hydrogen bonding with Gln221. Similarly, for LXRβ, compounds **1** and **2** interacted through hydrogen bonding with Thr316 and π–π stacking with Phe268. Compound **3** also exhibited a hydrogen bond with Thr316, together with multiple π–π interactions involving Phe329, Phe340, Trp457, and Arg305. These additional aromatic and polar interactions likely contribute to the stabilization of ligand–receptor complexes and complement the hydrogen bonding pattern previously described for flavonoid–LXR binding [36,40,41,42].

Docking scores further support these observations. As shown in Table 1, kaempferol glycosides displayed stronger binding energies (ΔG values between −9.7 and −10.5 kcal/mol) as compared to quercitrin and afzelin (−7.5 to −7.9 kcal/mol). These more negative scores indicate higher predicted affinities for both LXR isoforms, in line with the presence of multiple stabilizing interactions. The difference in hydrogen bond distance for kaempferol-3-O-(6″-*p*-coumaroylglucoside), which was weak for LXRα (>3.0 Å) but strong for LXRβ (<3.0 Å), may explain the slightly more favorable docking score observed with LXRβ. This highlights that not only the presence of a hydrogen bond but also the broader interaction network—hydrophobic, aromatic, and electrostatic—determines the overall binding strength [43].

Taken together, these results reinforce the role of glycosylated flavonols as potential LXR agonists. By combining canonical hydrogen bonding with Thr302/Thr316 and additional stabilizing π–π interactions, these compounds show binding energies comparable to those reported for other natural flavonoids with LXR activity [15,36]. This integrated analysis provides a stronger structural rationale for the observed in vitro upregulation of APOE and ABCA1, supporting the therapeutic potential of *Nectandra reticulata* metabolites in modulating lipid metabolism and neuroprotection (Table 3).

### 2.4. In Vitro Upregulation of LXR Target Genes by N. reticulata Extracted Compounds

Astrocytes play a pivotal role in the pathophysiology of Alzheimer’s disease (AD), as they are involved in glucose, lipid, and glutamate metabolism. They are the major brain cells responsible for the expression of *APOE* and *ABCA1* and have a central role in cholesterol metabolism and HDL biogenesis, processes that are closely associated with Aβ clearance. It is well known that the ε4 isoform of ApoE is the strongest genetic risk factor for late-onset AD (LOAD). In this study, we used U87 glioma cells, which are astrocyte-derived, express both *APOE* and *ABCA1*, and provide a suitable and reproducible model for preliminary investigations of LXR-mediated transcriptional regulation [44,45].

**Figure 6:**
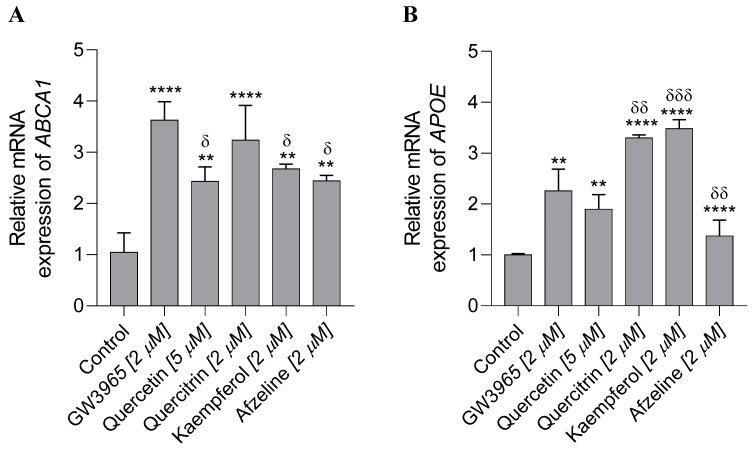
*APOE* and *ABCA1* mRNA levels. U87 cells were treated with a control (vehicle) and the flavonoid compounds. qRT-PCR were used to examine the mRNA levels, and fold changes were calculated by the 2-ΔΔCt method as compared with non-infection cell control and using endogenous GAPDH mRNA level for normalization. (**A**) The fold change of ABCA1 in mRNA levels; (**B**) the fold change of APOE in mRNA levels. The data are shown as the mean ± SE from three sets of independent experiments. Differences against the control group: ** *p* < 0.01; **** *p* < 0.0001. Differences against the GW3965 group: ^δ^ *p* < 0.05; ^δδ^ *p* < 0.01; ^δδδ^ *p* < 0.001.

Using this model, we found that treatment with 2 μM GW3965 (synthetic agonist of LXRs) increased the mRNA expression of both *APOE* (*p* < 0.001) and *ABCA1* (*p* < 0.0001) as compared to the vehicle treatment (Figure 6). Regarding the identified compounds, it was found that 2 μM quercitrin (compound **1**) increased the expression of both *APOE* (*p* < 0.0001) and *ABCA1* (*p* < 0.0001) as compared to the vehicle treatment (Figure 6). In addition, 5 μM quercetin (the hydrolyzed product of quercitrin) also increased the expression of both *APOE* (*p* < 0.05) and *ABCA1* (*p* < 0.05) (Figure 6). These results suggest that quercitrin is more active than its hydrolyzed counterpart, quercetin, suggesting that its glycosylation promotes its agonist activity. Our results are consistent with previous reports indicating that glycosides can modulate LXR affinity and selectivity through interactions involving both their sugar and aglycone moieties, thereby influencing receptor conformation and downstream transcriptional activity. The sugar component can affect binding interactions, while structural modifications to the aglycone may alter affinity and selectivity, for example, by strengthening hydrogen bond formation [46].

Regarding afzelin (compound **2**) and its hydrolyzed product kaempferol, 2 μM afzelin increased the expression of *ABCA1* (*p* < 0.01) without significantly changing ApoE levels as compared to the vehicle. In contrast, 2 μM kaempferol increased the expression of both *APOE* (*p* < 0.0001) and *ABCA1* (*p* < 0.001) (Figure 6), suggesting that kaempferol is more active in the absence of the glycosylated group.

When comparing the agonistic strength of the flavonoids with the synthetic agonist GW3965, we observed that 2 μM quercitrin elicited *ABCA1* mRNA expression comparable to that of 2 μM GW3965, while GW3965 was stronger (*p* < 0.05) than 5 μM quercetin, 2 μM afzelin, or 2 μM kaempferol. In contrast, for *APOE*, mRNA expression was similarly induced by 2 μM GW3965 and 5 μM quercetin, whereas 2 μM quercitrin (*p* < 0.01) and 2 μM kaempferol (*p* < 0.001) produced significantly higher expression levels than 2 μM GW3965. These findings suggest that quercitrin and kaempferol may act as particularly strong natural LXR agonists, with selective effects on APOE expression.

Finally, in this study we used sublethal concentrations of GW3965 and flavonoids (2–5 μM), which are relatively high as compared with typical plasma levels of dietary flavonoids that generally occur in the nanomolar to low micromolar range. However, tissue-specific accumulation and metabolism may lead to locally higher concentrations. Further studies using primary astrocytes, neuronal cultures, and in vivo models of AD will be required to validate and confirm the translational relevance of our findings.

## 3. Material and Methods

### 3.1. Chemicals and Standards

Luteolin was purchased from Fluka^®^ Analytical (Fisher scientific, Neuhofstrasse, Reinach, Switzerland); quercitrin (quercetin-3-*O*-rhamnoside) and kaempferol (China), *p*-coumaric acid (United Kingdom), and quercetin (India) were obtained from SIGMA. Afzelin (kaempferol-3-*O*-rhamnoside) was obtained from the extract of *N. reticulata* by traditional chromatographic techniques. All those standards, in solid state, had more than 95% (*w/w*) purity. Methanol and acetonitrile, both HPLC grade, were purchased from Honeywell (Muskegon, MI, USA). LiChropur HPLC-MS grade formic acid was purchased from Merck (Darmstadt, Germany). Supelco LiChrosolv HPLC-MS grade acetonitrile was purchased from Sigma-Aldrich (Darmstadt, Germany).

### 3.2. Plant Material and Extraction

*N. reticulata* leaves were collected in Granada (Cundinamarca, Colombia), 31 km from Bogotá D.C. A specimen of the species was deposited and identified by the Herbario Nacional de Colombia at Universidad Nacional de Colombia whose code is COL547368. Subsequently, leaves were dried at room temperature, ground to a particle size lower than 2 mm, extracted by percolation with 96% (*v*/*v*) aqueous ethanol, and rotavaporated at 40 °C.

### 3.3. Acid Hydrolysis of the Plant Extract

The plant extract (50 mg) was hydrolyzed by reflux at 80 °C for 2 h, using 5 mL of 1.2 M HCl and 2 mg of ascorbic acid in a hydroalcoholic methanol mixture [31]. In addition, solubility tests were carried out modifying the proportion of methanol in the reaction solvent, testing aqueous solutions of methanol at 50, 75 or 80% (*v*/*v*). After the reaction time was completed, the mixture was cooled to room temperature, the pH was set to 4–6, and the product was dried and re-suspended up to 10.0 mL with 100% (*v*/*v*) methanol in a volumetric flask. Finally, the hydrolysis reaction product was filtered using a 0.45 µm filter and analyzed by RP-UHPLC-DAD.

### 3.4. Identification of Metabolites

The samples (crude extract and hydrolysis reaction products) were analyzed on a Dionexin vit Ultimate 3000 system (Thermo Scientific, San Jose, CA, USA). The system was equipped with a pump, an autosampler, and a photodiode array detector (DAD). The stationary phase was a Hypersyl Gold RP column (Thermo Scientific; 150 mm × 2.1 mm id; 1.9 µm particle size), operated at 40 °C. The eluents were water/formic acid (99.9/0.1 *v*/*v*) (eluent A) and acetonitrile (eluent B). The elution program was 0–4 min, linear gradient from 5 to 20% B; 4–10 min, isocratic at 20% B; 10–12 min, linear gradient from 20 to 25% B; 12–16 min, isocratic at 25% B; 16–22 min, linear gradient from 25 to 52% B; 22–26 min, isocratic at 52% B; 26–30 min, linear gradient from 52 to 70% B; 30–33 min, linear gradient from 70 to 100% B; 33–36 min, isocratic at 100% B; 36–41 min, linear gradient from 100 to 5% B; 41–46 min, isocratic at 5% B. The flow rate was 400 μL/min with an injection volume of 5 μL.

In addition, the crude extract was analyzed on an Agilent Technologies 1260 liquid chromatography system coupled to a Q-TOF 6545 quadrupole time-of-flight mass analyzer with electrospray ionization. For the chromatographic separation, the same conditions of the previously described program were used; only the detection system was changed. Mass spectrometry detection was performed in full scan in both positive and negative ESI mode from 100 to 1100 *m*/*z*, with a source voltage of 3000 V and an ion transfer tube temperature of 290 °C.

The identification was based on the analysis of retention time and the elution order, UV-Vis spectra, HR-MS/MS data, reaction products after acidic hydrolysis, comparison of chromatographic, spectroscopic, and spectrometric features of chromatographic peaks with standards, and comparison with the literature.

### 3.5. Molecular Docking

The identified compounds were tested for their ability to bind to either LXRα or LXRβ by molecular docking, using AutoDock Vina (ADV, v.1.2). The 3D structure of LXRα and LXRβ (PDB ID: 1PQ6 and 3IPQ, respectively) was obtained from the Protein Data Bank. The pdbqt files were prepared by removing both water molecules and all ligands included in the crystal, and only polar hydrogen atoms were set. Visualization of the binding mode predictions was performed with UCSF Chimera 1.15 molecular viewer. Next, a docking grid box with a size of 25 × 25 × 25 Å was created. A molecular re-docking of GW3965 into LXRα and LXRβ (1PQ6 and 3IPQ) was performed to validate the methodology previously described. In addition, a molecular docking of active compounds quercetin and apigenin was conducted as part of the validation studies [32]. Subsequently, molecular docking analysis was conducted on the molecules identified from the extract and interactions between ligands, and nuclear receptors were analyzed.

### 3.6. In Vitro Upregulation of LXR Target Genes by N. reticulata Extracted Compounds

To corroborate the LXR agonistic action of pure compounds extracted from *Nectandra reticulata,* the expression of both *APOE* and *ABCA1* genes in the U87 cell line after treatment with the control (vehicle), the synthetic agonist of LXRs (GW3965) as positive control, and compounds from *Nectandra reticulata* were evaluated. Induction of *APOE* mRNA in U87 cells has previously been used to study nuclear receptor activation [16].

### 3.7. Cell Culture and Viability

Human U87 cell line (human glioma) was grown in Dulbecco’s Modified Eagle’s Medium (DMEM; Invitrogen) supplemented with 10% fetal bovine serum (FBS, Biowest) and 1% (*w/v*) penicillin–streptomycin (Santa Cruz Biotechnology, Inc., Heidelberg, Germany) in a humidified atmosphere at 37 °C in 5% CO_2_.

### 3.8. Real-Time Reverse Transcription-Polymerase Chain Reaction (qRT-PCR)

To monitor the in vitro gene expression of LXR target genes, qRT-PCR was performed. Briefly, total RNA from U87 cells was extracted using the RNA Easy Kit (Qiagen, Hilden, Germany) according to the manufacturer’s instructions. The RNA was quantified using the NanoDropTM 2000 (Thermo Scientific, San Jose, CA, USA). For RNA detection and quantitation, the Luna^®^ Universal One-Step qRT-PCR kit (E3005, New England Biolabs, Ipswich, MA, USA) was used according to the manufacturer’s protocol. Primer pairs (Microgen, Republic of Korea) for *APOE* (forward CCTCAAGAGCTGGTTCGAG, reverse TCGGCGTTCAGTGATTGTC), *ABCA1* (forward GCTTTCAATCATCCCCTGAA, reverse CAGGTGTT TGCTTTGCTGA), and *GADPF* (forward GACCTGCCGTCTAGAAAAACC, reverse ACCACCTGGTGCTCAGTGTAG) were designed to hybridize to unique regions of the appropriate gene sequence. Statistical significance was determined using a one-way ANOVA followed by Tukey’s post hoc test. Data are expressed as means ± standard error of the mean (SEM), with n = 3 biological replicates.

## 4. Conclusions

We report the flavonoid composition of an ethanolic extract of *Nectandra reticulata*. The main compound corresponds to quercitrin; the other two compounds are afzelin and either kaempferol 3-*O*- or 7-*O*-(6″-*p*-coumaroyl glucoside). By molecular docking, it was possible to identify that quercitrin, afzelin, and kaempferol-7-*O*-(6″-*p*-coumaroylglucoside) are capable of binding to the amino acids threonine 302 and threonine 316 (LXRα and LXRβ, respectively), which are related to the LXR agonist activity. The studied flavonoids increased the expression of mRNA of both *APOE* and *ABCA1* target genes of LXRs, as observed by qRT-PCR. All in all, they could be related to the agonistic activity of the ethanolic extract of *Nectandra reticulata*. It would be important to evaluate the activity of these isolated interests and verify whether they have antagonistic, additive, or synergistic interaction. The flavonoids isolated and characterized in this study as LXR agonists exhibit the potential to modulate lipid homeostasis and regulate inflammatory pathways. These results indicate that the compounds are promising candidates for subsequent evaluation in animal models to confirm their biological activity and facilitate the advancement of more specific pharmacological studies.

## Figures and Tables

**Table 1:** Identification of markers in the ethanolic extract by UHPLC-DAD and UHPLC-ESI-HR-MS/MS.

Peak No.	Retention Time (min)		UVmax λ (nm)	MS	MS^2^	Molecular Formula	AME * (ppm)	Tentative Annotation
1	7.80		260; 349	447.0936	300.0273	C21H20O11	1.8	Quercitrin
				[M−H]^−^	[M-rhamnoseH]^−●^			
				303.0509				
	[M-rhamnose+H]^+^							
2	9.44		266; 341	431.0980	284.0323	C21H20O10	−0.5	Afzelin
				[M−H]^−^	[M-rhamnose-H]^−●^			
				287.0561				
	[M-rhamnose+H]^+^							
3	14.26		229; 268; 314	593.1296	285.0396	C30H26O13	−0.2	Kaempferol 3-(6″-*p*- coumaroylglucoside) or Kaempferol 7-(6″-*p*-coumaroylglucoside)
				[M−H]^−^	[M-coumaroylglucoside-H]^−^			
				595.1464	309.0981			
				[M+H]^+^	[M-kaempferol+H]^+^			
					287.0559			
					[M-coumaroylglucoside+H]^+^			

* AME: accurate mass error (ppm).

**Table 2:** Aglycones present in the extract after hydrolysis.

Compound	Standard Retention Time (min)	Standard UV λ (nm)	Hydrolyzed Extract Retention Time (min)	UV λ (nm)
Quercetin	12.30	202; 257; 368	12.29	203; 257; 368
Kaempferol	16.59	197; 267; 367	16.57	197; 267; 366
Luteolin	12.63	208; 256; 348	12.54 *	230; 310 *
*p*-Coumaric acid	6.15	227; 310	6.16	229; 311

* There is no match between the standard and the reaction product.

**Table 3:** Predicted binding energies (ΔG, kcal/mol) of selected compounds to LXRα and LXRβ receptors. The values were calculated using AutoDock Vina software.

Compound	LXRα Binding Energy (Δ*G*, kcal/mol)	LXRβ Binding Energy (Δ*G,* kcal/mol)
Quercitrin (**1**)	−7.5	−7.5
Afzelin (**2**)	−7.5	−7.9
Kaempferol 7-*O*-(6″-*p*-coumaroylglucoside) (**3**)	−9.9	−9.7
Kaempferol 3-*O*-(6″-*p*-coumaroylglucoside) (**3**)	−10.3	−10.5

## Data Availability

The data presented in this study are openly available in https://repositorio.unal.edu.co/items/75fa9d72-de03-4d0e-b356-8cd37bd8ee9a and https://repositorio.unal.edu.co/bitstreams/a442c82f-3471-458a-ab1f-9fc359745d9a/download (accessed on 11 October 2025).

## Abbreviations

The following abbreviations are used in this manuscript:

LXRs	Liver X Receptors
ApoE	Apolipoprotein E
ABCA1	ABC Transporter Protein Member 1
AD	Alzheimer Diseases
RP	Reversed Phase
UHPLC	Ultra High Performance Liquid Chromatography
DAD	Diode Array Detector
ESI-HR-MS	High Resolution Mass Spectrometry with Electrospray ionization
qRT-PCR	Real Time Reverse Transcription Polymerase Chain Reaction
RNA	Ribonucleic Acid
mRNA	Messenger Ribonucleic Acid
LXRα	Liver X Receptors Alpha
LXRβ	Liver X Receptors Beta
a.m.u.	Atomic Mass Unit
HPLC	High Performance Liquid Chromatography
HCl	Hydrochloric Acid
Q-TOF	Quadrupole Time of Flight
